# Dysbiosis of oral microbiota and its association with salivary immunological biomarkers in autoimmune liver disease

**DOI:** 10.1371/journal.pone.0198757

**Published:** 2018-07-03

**Authors:** Kazumichi Abe, Atsushi Takahashi, Masashi Fujita, Hiromichi Imaizumi, Manabu Hayashi, Ken Okai, Hiromasa Ohira

**Affiliations:** 1 Department of Gastroenterology, Fukushima Medical University School of Medicine, Fukushima, Japan; 2 Department of Internal Medicine, Hanawa Kosei Hospital, Higashishirakawa, Japan; Laval University, CANADA

## Abstract

The gut microbiota has recently been recognized to play a role in the pathogenesis of autoimmune liver disease (AILD), mainly primary biliary cholangitis (PBC) and autoimmune hepatitis (AIH). This study aimed to analyze and compare the composition of the oral microbiota of 56 patients with AILD and 15 healthy controls (HCs) and to evaluate its association with salivary immunological biomarkers and gut microbiota. The subjects included 39 patients with PBC and 17 patients with AIH diagnosed at our hospital. The control population comprised 15 matched HCs. Salivary and fecal samples were collected for analysis of the microbiome by terminal restriction fragment length polymorphism of 16S rDNA. Correlations between immunological biomarkers measured by Bio-Plex assay (Bio-Rad) and the oral microbiomes of patients with PBC and AIH were assessed. Patients with AIH showed a significant increase in *Veillonella* with a concurrent decrease in *Streptococcus* in the oral microbiota compared with the HCs. Patients with PBC showed significant increases in *Eubacterium* and *Veillonella* and a significant decrease in *Fusobacterium* in the oral microbiota compared with the HCs. Immunological biomarker analysis showed elevated levels of inflammatory cytokines (IL-1β, IFN-γ, TNF-α, IL-8) and immunoglobulin A in the saliva of patients with AILD. The relative abundance of *Veillonella* was positively correlated with the levels of IL-1β, IL-8 and immunoglobulin A in saliva and the relative abundance of *Lactobacillales* in feces. Dysbiosis of the oral microbiota is associated with inflammatory responses and reflects changes in the gut microbiota of patients with AILD. Dysbiosis may play an important role in the pathogenesis of AILD.

## Introduction

Primary biliary cholangitis (PBC) and autoimmune hepatitis (AIH) are classically viewed as distinct autoimmune liver diseases (AILDs). PBC is a progressive AILD characterized by portal inflammation, immune-mediated destruction of the intrahepatic bile ducts, and the presence of highly specific anti-mitochondrial antibodies in serum [1,2]. AIH manifests as chronic liver inflammation of an unknown cause. It generally affects young to middle-aged females and is associated with the presence of autoantibodies and hypergammaglobulinemia [3]. AILD is thought to be triggered by environmental factors in genetically susceptible individuals. Genome-wide association and murine model studies have expanded our knowledge of AILD; however, the pathogenesis of the disease remains obscure.

The oral cavity is a large reservoir of bacteria of more than 700 species or phylotypes and is profoundly relevant to host health and disease [4–6]. The role of oral and gut microbiota in the pathogenesis of immune-related diseases has been highlighted in autoimmune diseases, such as autoimmune encephalomyelitis, rheumatoid arthritis, and inflammatory bowel disease [7–13]. A previous report revealed that there was evidence of pervasive immune-microbiota interface changes in the saliva of patients with cirrhosis similar to that found in stool [14]. Recently, culture-independent techniques have revolutionized the knowledge of the gut and oral microbiota. These techniques are based on sequence divergences of the small subunit ribosomal ribonucleic acid (16S rRNA) and can demonstrate the microbial diversity of the gut and oral microbiota, providing qualitative as well as quantitative information on bacterial species and changes in the gut and oral microbiota in health and disease.

It is increasingly recognized that the composition of the gut microbiota plays a critical role in influencing the predisposition to PBC and AIH [15–20]. However, direct evaluation of the oral microbiome has not been performed in AILD. This study aimed to analyze and compare the composition of the salivary microbiota between patients with AILD and healthy controls (HCs) and to evaluate its association with oral immunological biomarkers.

## Materials and methods

### Study population

This study included 39 patients with PBC and 17 with AIH who received a diagnosis at Fukushima Medical University Hospital and Hanawa Kosei Hospital between 1996 and 2016, as well as 15 HCs. As HCs, normal serum was collected from staff members and their families in our department. The diagnosis of AIH was based on the revised and simplified International Autoimmune Hepatitis Group (IAIHG) scoring system [21–23]. Patients with other causes of chronic liver disease, particularly alcohol abuse, chronic hepatitis B, or hepatitis C, were excluded from the AILD patient group. Patients were diagnosed as having PBC features if they met at least two of the following three criteria: 1) chronic elevation of cholestatic liver enzymes alkaline phosphatase (ALP) and gamma-glutamyl transpeptidase (γGTP) for at least six months; 2) presence of serum anti-mitochondrial antibody (AMA) detected by either indirect immunofluorescence or ELISA using commercially available kits; and 3) typical histological findings from biopsied liver specimens [24]. Twenty-nine patients with PBC had liver biopsies.

The data used for analysis included patient background parameters (age, sex, observation period, body mass index (BMI)), clinical parameters at sample collection (aspartate aminotransferase (AST), alanine transaminase (ALT), ALP, γGTP, total bilirubin (TB), IgG, IgM, anti-nuclear antibodies [ANA], AMA, fibrosis (FIB)-4 index), histological parameters at presentation (Scheuer stage for PBC, Fibrosis for AIH) and therapeutic methods. The histological findings of PBC were graded according to the Scheuer staging system [25]. The fibrosis stage of AIH was evaluated according to the METAVIR scoring system [26] and graded as follows: F0, no fibrosis; F1, stellate enlargement of portal tracts without spectrum formation; F2, enlargement of portal tracts with rare spectrum formation; F3, numerous septa without cirrhosis; and F4, cirrhosis. Nine patients with AIH (53%) and 11 patients with PBC (28%) were concomitantly using proton pump inhibitors (PPIs). Sjögren’s syndrome was associated with 2 cases of AIH and 1 case of PBC. The patients with AIH were classified as the normal liver function group (AST and ALT ≤33 U/L) and abnormal liver function group (AST or ALT >33 U/L). The patients with PBC were classified as the normal liver function group (ALP ≤359 U/L and γGTP ≤50 U/L) and abnormal liver function group (ALP >359 U/L or γGTP >50 U/L). Exclusion criteria were as follows: (i) antibiotic use within the past 3 months; (ii) otolaryngology consultation due to sinusitis, tonsillitis or tonsilloliths within the past 3 months; (iii) use of gargling solution on the day of screening; and (iv) periodontitis.

### Sample collection and DNA extraction

All subjects underwent stool and saliva collection on the same day. Unstimulated saliva samples collected from subjects were immediately stored at -20°C until use. The saliva samples were homogenized with zirconia beads in a 2.0-mL screw cap tube by FastPrep 24 Instrument (MP Biomedicals, Santa Ana, CA) at 5 m/s for 90 sec. DNAs were extracted from 100 μL of the saliva and purified with the MORA-EXTRACT DNA extraction kit (Kyokuto Pharmaceuticals, Tokyo, Japan) in accordance with the manufacturer's instructions. The DNAs were eluted with 100 μL of TE (10 mM Tris-HCl, 1 mM EDTA, pH 8.0).

Fecal samples were immediately suspended in a solution containing 100 mM Tris-HCl (pH 9.0), 40 mM Tris-EDTA (pH 8.0), 4 M guanidine thiocyanate, and 0.001% bromothymol blue. An aliquot of 1.2 mL of the suspension was homogenized with zirconia beads in a 2.0-ml screw cap tube by FastPrep 24 Instrument (MP Biomedicals) at 5 m/s for 2 min and placed on ice for 1 min. After centrifugation at 5000 × g for 1 min, DNA was extracted from 200 μL of the suspension using an automatic nucleic acid extractor (Precision System Science, Chiba, Japan). MagDEA DNA 200 (GC) (Precision System Science) was used as the reagent for automatic nucleic acid extraction [27, 28].

### Terminal restriction fragment length polymorphism (T-RFLP)

T-RFLP analyses were performed by TechnoSuruga Laboratory (Shizuoka, Japan). T-RFLP analyses for salivary samples were performed as previously described [29]. The primers used for the PCR amplification of 16S rRNA gene sequences were 27F (5’-AGAGTTTGATCCTGGCTCAG-3’) and 1492R (5’-GGTTACCTTGTTACGA-CTT-3’). Primer 27F was labeled at the 5’ end with 6- carboxyfluorescein (6-FAM), which was synthesized by Thermo Fisher Scientific. For the 16S rDNA amplified from human saliva-extracted DNA, HotStarTaq DNA Polymerase (QIAGEN, Hilden, Germany) by Thermal Cycler Dice (Takara, Shiga, Japan) was used. The amplification program was as follows: preheating at 94°C for 15 min, 30 cycles of denaturation at 94°C for 30 s, annealing at 50°C for 30 s, extension at 72°C for 2 min, and finally, a terminal extension at 72°C for 10 min. Amplified DNA was verified by the electrophoresis of PCR mixture aliquots (2 μL) in 1.0% agarose in TAE buffer. The amplified DNA was purified by a MultiScreen PCR 96 Filter Plate (Millipore, Billerica, MA).

The purified PCR product (3 μL) was digested with 10 U of Fast Digest *Msp*I (Thermo Fisher Scientific) in a total volume of 15 μL at 37°C for 10 min. The restriction digestion products (0.5 μL) were mixed with 10 μL of deionized formamide and 0.5 μL of DNA fragment length standard. The standard size marker was MapMarker X-Rhodamine Labeled 50–1000 bp (BioVentures, Murfreesboro, TN). The samples were denatured at 95°C for 2 min and then placed immediately on ice. The length of T-RF was determined on an ABI PRISM 3130xl Genetic Analyzer (Thermo Fisher Scientific), and the length and peak area were determined using the genotype software GeneMapper (Thermo Fisher Scientific). Fragment sizes were estimated using the Local Southern method in GeneMapper software (Thermo Fisher Scientific). T-RFs with a peak height of less than 50 fluorescence units were excluded from the analysis. Fragments were resolved to one base pair by manual alignment of the size standard peaks from different electropherograms. Predicted T-RFLP patterns of the 16S rDNAs of known bacterial species were obtained using the sequence [29].

T-RFLP analyses for fecal samples were performed as previously described [30, 31]. The 16S rRNA sequences were amplified from human fecal DNA by using a fluorescently labeled 516F primer (5’-TGCCAGCAGCCGCGGTA-3’; *E*. *coli* positions 516–532) and 1510R primer (5’-GGTTACCTTGTTACGACTT-3’; E. coli positions 1510–1492). The 5’-ends of the forward primers were labeled with 6 -carboxyfluorescein (6-FAM), which was synthesized by Thermo Fisher Scientific. The PCR amplifications of DNA samples (10 ng of each DNA) were performed according to a protocol described by Nagashima et al. The purified PCR products (2 μL) were digested with 10 U of Fast Digest *Bsl*I (Thermo Fisher Scientific) at 37°C for 10 min. The length of the T-RF fragment was determined with an ABI PRISM 3130xl Genetic Analyzer (Thermo Fisher Scientific). The standard size marker was MapMarker X-Rhodamine Labeled 50–1000 bp (BioVentures). The T-RFs were divided into 29 operational taxonomic units (OTUs). The OTUs were quantified as the percentage of individual OTU per total OTU areas, which were expressed as the percentage of the area under the curve (% AUC). The bacteria were predicted for each classification unit, and the corresponding OTU was identified according to reference Human Fecal Microbiota T-RFLP profiling (https://www.tecsrg.co.jp/t-rflp/t_rflp_hito_OTU.html).

### Immunoassays

Concentrations of 17 (IL-1β, IL-2, IL-4, IL-5, IL-6, IL-7, IL-8, IL-10, IL-12p70, IL-13, IL-17, TNF-α, MCP-1, MIP-1β, IFN-γ, G-CSF, GM-CSF) cytokines in serum and saliva were analyzed using a Luminex Bio-Plex 200 system (Bio-Rad, Hercules, CA, USA) according to the manufacturer’s protocol. IgA levels were measured using an ELISA kit (Yanaihara, Shizuoka, Japan). Salivary lysozyme levels were measured using an ELISA kit (CUSABIO, Hubei, China).

### Statistical analysis

The results are expressed as the mean ± SD. The Mann-Whitney U-test was used to compare the bacterial abundance or cytokine levels between the HC and AILD groups. Correlations between the bacterial abundance and immunological markers in saliva or the bacterial abundance in feces were assessed using Spearman’s rank correlation coefficient. The difference in the ratio of bacterial groups was examined by the χ^2^ test. Shannon diversity indices were used to compare the diversity of the T-RFLP profiles between HC and AILD groups. The T-RFLP profiles were clustered by hierarchical cluster analysis and analyzed by principal component analysis (PCA). Univariate and multivariate logistic regression analyses were used to assess microbiomes associated with AILD patients. All statistical analyses were performed using Prism 6.0 software (GraphPad Software, Inc.) and JMP pro 13.1 (SAS Institute Inc., Cary, NC, U.S.A.). *P*<0.05 was considered significant.

### Ethics statement

The study was approved by the ethics committee of Fukushima Medical University School of Medicine. Written informed consent was obtained from all subjects.

## Results

### Clinical characteristics of HCs and patients with PBC or AIH

Table 1 shows the characteristics of the matched HCs and the patients with PBC or AIH. Patients with PBC (mean age, 63 years; male:female ratio, 5:34) had an ALT level of 27 ± 16 U/L, an ALP level of 321 ± 111 U/L, and a γGTP level of 59 ± 44 U/l. In all, 26 patients were treated with ursodeoxycholic acid (UDCA), and 11 were treated with UDCA and bezafibrate. Patients with AIH (mean age, 60 years; male:female ratio, 2:15) had an ALT level of 19 ± 10 U/L and an IgG level of 1473 ± 821 mg/dl; 11 patients were treated with prednisolone, and 4 were treated with prednisolone and azathioprine. No significant differences were found between the PBC and AIH groups with respect to age, sex or BMI.

**Table 1:** Clinical characteristics of HCs and patients with PBC or AIH.

	PBC	AIH	HC
	n = 39	n = 17	n = 15
Age, years (mean±sd)	63 ± 12	60 ± 11	58 ± 10
Gender, female (%)	34 (87.2%)	15 (88.2%)	13 (86.7%)
BMI, kg/m^2^ (mean±sd)	23.1 ± 2.8	22.7 ± 3.5	23.2±1.6
AST, U/L, (mean±sd)	32 ± 14	23 ± 6	19 ± 4
ALT, U/L, (mean±sd)	27 ± 17	19 ± 11	16 ± 4
ALP, U/L, (mean±sd)	321 ± 112	200 ± 60	NA
γGTP U/L, (mean±sd)	59 ± 44	20 ± 8	20 ± 8
TB, mg/dL, (mean±sd)	0.8 ± 0.3	0.9 ± 0.3	NA
IgM, mg/dL, (mean±sd)	226 ± 144	NA	NA
IgG, mg/dL, (mean±sd)	1519 ± 260	1535 ± 855	NA
ANA, ±, n (+%)	28/11 (71.8%)	16/1 (94.1%)	NA
AMA, ±, n (+%)	36/3 (92.3%)	3/14 (17.6%)	NA
FIB-4, (mean±sd)	2.0 ± 1.1	2.2 ± 1.4	NA
Scheuer 1/2/3/4 /Fibrosis 0/1/2/3/4	22/3/2/2	1/10/1/4/1	NA
UDCA, ±, n (+%)	37/2 (94.9%)	14/3 (82.4%)	NA
BF, ±, n (+%)	11/28 (28.2%)	NA	NA
PSL, ±, n (+%)	1/38 (2.6%)	15/2 (88.2%)	NA
Duration of disease, years (mean±sd)	7.5 ± 5.0	9.4 ± 8.6	NA

BMI, body mass index; ANA, anti-nuclear antibody; AMA, anti-mitochondrial antibody; FIB-4, fibrosis 4; UDCA, ursodeoxycholic acid; BF, bezafibrate; PSL, prednisolone; NA, not available

### Analysis of the salivary microbiota of the PBC, AIH and HC groups based on the T-RFLP profiles

**Fig 1:**
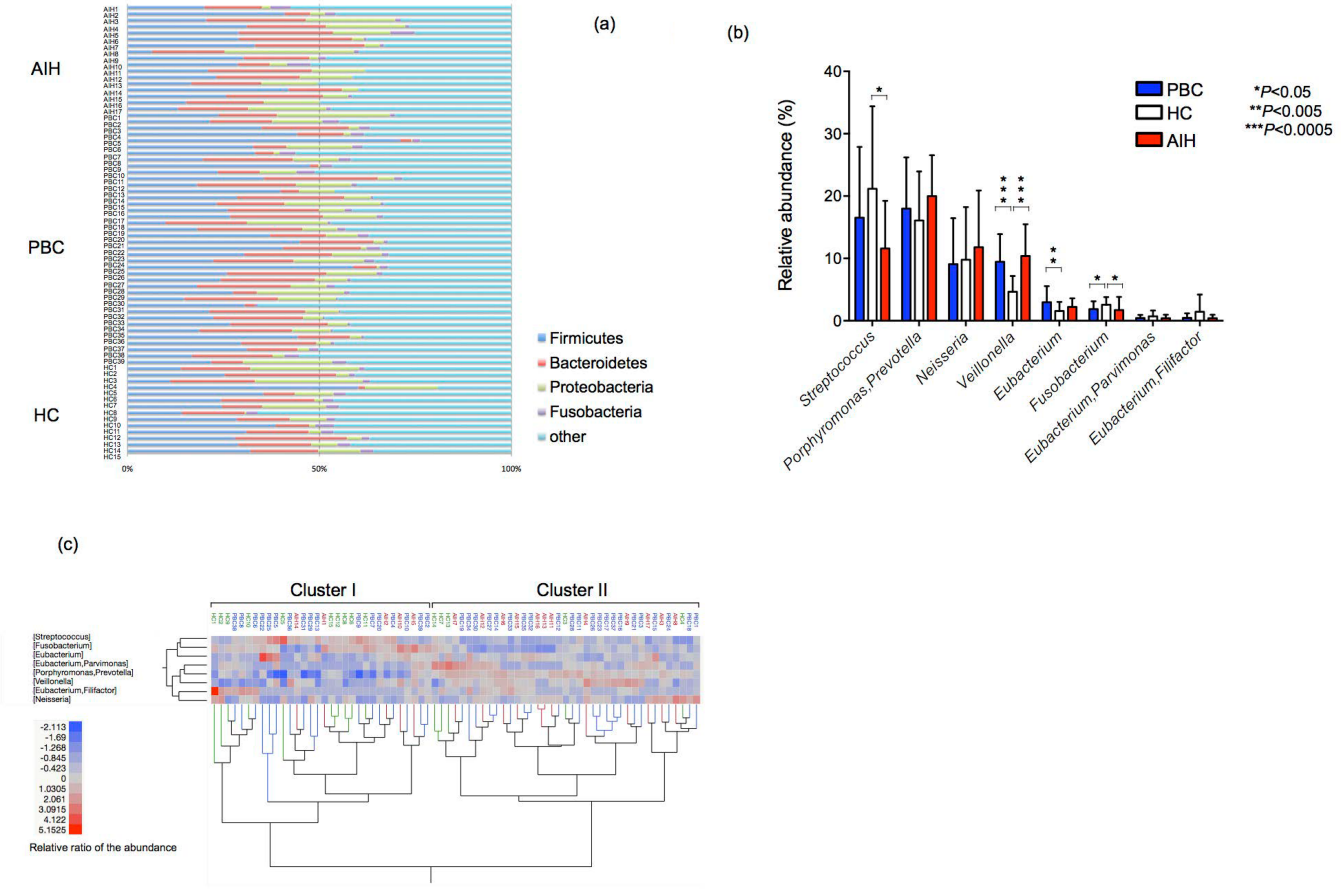
Analysis of the oral microbiota of the PBC, AIH and HC groups based on the T-RFLP profiles. (a) Bacterial composition at the phylum level. The relative abundance of the bacterial composition at the phylum level in each sample from the subjects in the AIH, PBC and HC groups is shown on a bar chart. The ID of each subject is tagged to the left of the bar. AIH, autoimmune hepatitis; PBC, primary biliary cholangitis; HC, healthy control. (b) Mean genus abundance in the PBC, AIH and HC groups. Plotted values are the mean abundance of the 8 abundant genera in each group. The results are expressed as the mean ± SD. Differences were compared using the Mann-Whitney U-test; *P<0.05, **P<0.01, ***P<0.0005. (c) Cluster analysis of the bacterial compositions in the saliva samples. The samples from 71 subjects and 8 dominant genera are represented on a double-hierarchical clustering heat map. The blue and red squares represent lower and higher abundances, respectively. The clusters at the bottom indicate similarities among the individual (IDs on the top side) profiles at the genus level. The bacterial compositions are classified into two clusters, Cluster I (n = 32) and Cluster II (n = 39). The clusters on the left side indicate the genera showing similarity in the frequency of identification among samples.

Fig 1A shows the relative abundance of the bacterial composition at the phylum level in each sample from subjects in the AIH, PBC and HC groups. The most dominant phylum was *Firmicutes* in the AIH, PBC and HC groups. Indeed, the average relative abundance of phyla *Firmicutes* in the AIH, PBC and HC groups was 25.1%, 29.8% and 27.8%, respectively. No significant difference at the phylum level in *Firmicutes*, *Bacteroidetes and Proteobacteria* were observed among the groups. Analysis at the phylum level showed that the relative abundance of *Fusobacteria* was significantly lower in both the AIH and PBC groups than in the HC group (*P*<0.05).

T-RFLP analysis of the salivary microbiota in all 71 subjects revealed 78 peaks by digestion with *MspI*. The relative amounts of several T-RFs in the AIH and PBC groups were significantly different from those in the HC group. When T-RFs were digested by *MspI*, there was a significantly higher frequency of genus *Veillonella* (OTU301) and genus *Eubacterium* (OTU166) and a lower frequency of genus *Fusobacterium* (OTU283) in the PBC group than in the HC group (Fig 1B). Moreover, there was a significantly higher frequency of genus *Veillonella* and a lower frequency of genus *Streptococcus* (OUT556, 563) and genus *Fusobacterium* in the AIH group than in the HC group. The oral microbiota of PPI users was not significantly different from that of non-PPI users among patients with AIH or PBC (S1 Fig). Fig 1C shows the cluster analysis based on the T-RFLP profiles of the salivary microbiota. The microbiota was classified into two groups: 32 subjects in Cluster I and 39 subjects in Cluster II. The majority of subjects in the AILD group (34/56, 60.7%) had a microbiota in Cluster II, while the majority of those in the HC group (10/15, 66.7%) had a microbiota in Cluster I (*P*<0.05, χ^2^ test).

### Cytokine levels in the saliva of HCs and patients with PBC or AIH

**Fig 2:**
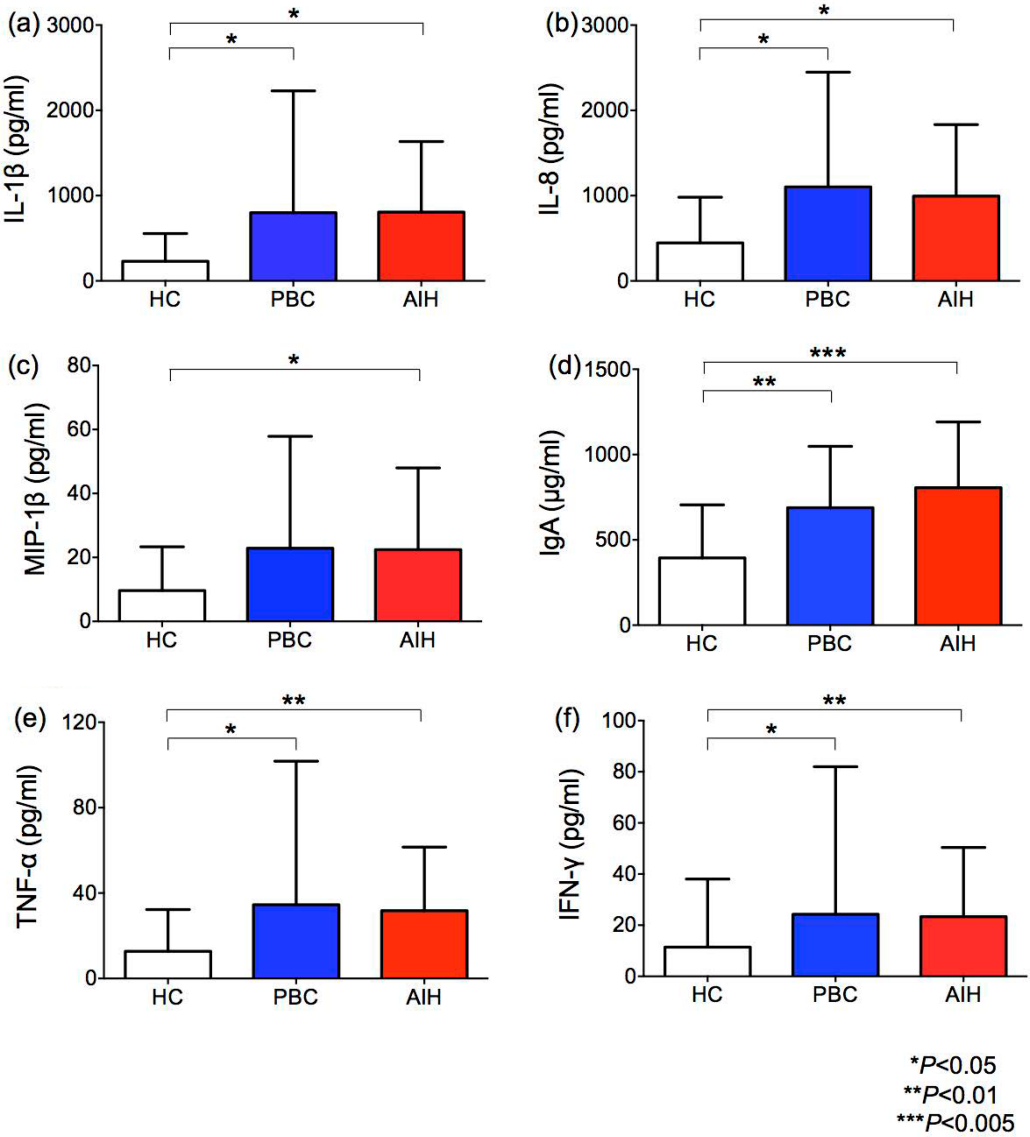
Cytokine levels in the saliva of HCs and patients with PBC or AIH. The salivary levels of IL-1β (a), IL-8 (b), MIP-1β (c), IgA (d), TNF-α (e), and IFN-γ (f). The results are expressed as the mean ± SD. Differences were compared using the Mann-Whitney U-test; *P<0.05, **P<0.01, ***P<0.005.

Given these changes in the salivary microbiota, we subsequently enrolled patients with AIH or PBC and age-matched HCs to study the inflammatory milieu in the saliva (Fig 2). None of the HCs were on PPIs or had diabetes or other chronic diseases. We found a significantly higher inflammatory response in AILD patients than in HCs, as shown by significantly higher IL-1β, IL-8 TNF-α, IFN-γ, MIP-1β and secretory IgA levels. No differences were observed in oral inflammatory markers between patients with/without PPI use (S1 Fig). In most samples, IL-2, IL-5, IL-10, IL-13, and GM-CSF were undetectable. There were no differences in the level of IL-4, IL-6, IL-7, IL-12p70, IL-17, G-CSF, MCP-1, or lysozyme between AILD patients and HCs (S1 Table).

### Correlation between the relative abundance of predominant genera and the level of immunological biomarkers in the saliva of AILD patients

We searched for correlations between the relative abundance of dominant bacterial genera and the measured biomarkers in the saliva of 56 patients with AILD. The results are shown in Table 2. The relative abundance of *Streptococcus* negatively correlated with the levels of IL-1β, IL-4, IL-6, IL-7, IL-8, IL-12p70, IL-17, G-CSF, IFN-γ and TNF-α, while the relative abundance of *Veillonella* and *Prevotella/Porphyromonas* (OTU93) positively correlated with the IgA level in the saliva of patients with PBC. Moreover, the relative abundance of *Neisseria* and *Eubacterium/Filifactor* (OTU490) positively correlated with the salivary cytokine levels of patients with PBC. On the other hand, the abundance of *Veillonella* positively correlated with the salivary levels of IL-1β, IL-6, IL-8, IL-12p70 and IgA of patients with AIH. Moreover, the relative abundance of *Eubacterium/Parvimonas* (OTU296) and *Eubacterium/Filifactor* positively correlated with the salivary cytokine levels of patients with AIH.

**Table 2:** Correlation between the relative abundance of predominant genera and the levels of immunological biomarkers in the saliva of AILD patients.

PBC group		IL-1β	IL-4	IL-6	IL-7	IL-8	IL-12p70	IL-13	IL-17	G-CSF	IFN-γ	MCP-1	MIP-1β	TNF-α	IgA	Lysozyme
*Streptococcus*	*r*	-0.5138	-0.3823	-0.4563	-0.4609	-0.5088	-0.3354	-0.2272	-0.3544	-0.595	-0.4192	-0.4011	-0.3097	-0.4105	-0.2461	0.2307
	*p*	0.0011**	0.0196*	0.0045**	0.0041**	0.0013**	0.0425*	0.1763	0.0314	0.0001***	0.0098**	0.0139*	0.0622	0.0116*	0.1421	0.1696
*Porphyromonas*,*Prevotella*	*r*	0.3487	0.1655	0.228	0.2235	0.2817	0.1278	0.006842	0.1243	0.3402	0.2158	0.3189	0.07406	0.2202	0.462	-0.2342
	*p*	0.0344*	0.3276	0.1748	0.1837	0.0913	0.4511	0.9679	0.4636	0.0394*	0.1996	0.0544	0.6631	0.1903	0.004**	0.1629
*Neisseria*	*r*	0.3402	0.1068	0.2956	0.1878	0.3172	0.1511	-0.1936	0.09596	0.4975	0.1331	0.1252	0.2111	0.1215	0.0651	-0.2788
	*p*	0.0394*	0.5294	0.0757	0.2657	0.0557	0.3722	0.251	0.5721	0.0017**	0.4324	0.4604	0.2097	0.4736	0.7019	0.0947
*Veillonella*	*r*	0.1584	0.2374	0.2498	0.1417	0.08653	0.1438	0.189	0.2036	0.08915	0.2367	0.2729	0.1396	0.2444	0.3788	0.2352
	*p*	0.3492	0.1572	0.136	0.403	0.6106	0.3958	0.2626	0.2269	0.5998	0.1584	0.1022	0.41	0.1449	0.0208*	0.1612
*Eubacterium*	*r*	-0.229	-0.1756	-0.1201	-0.05631	-0.1759	-0.2104	-0.02694	-0.1119	-0.06971	-0.1892	-0.1145	-0.1053	-0.2043	0.04506	0.02703
	*p*	0.1727	0.2984	0.4789	0.7406	0.2977	0.2113	0.8742	0.5096	0.6818	0.2621	0.4998	0.5352	0.2252	0.7911	0.8738
*Fusobacterium*	*r*	-0.233	-0.05708	-0.02407	-0.07125	-0.2105	-0.05558	-0.08552	-0.1122	-0.13	-0.09626	-0.02916	-0.07252	-0.1321	-0.1833	0.07492
	*p*	0.1651	0.7372	0.8876	0.6752	0.211	0.7439	0.6148	0.5087	0.443	0.5709	0.864	0.6697	0.4358	0.2774	0.6594
*Eubacterium*,*Parvimonas*	*r*	-0.1688	-0.09927	-0.03485	0.01166	-0.1811	-0.1075	0.09426	-0.05702	-0.1292	-0.06646	0.04122	-0.1408	-0.07658	0.1103	0.05913
	*p*	0.3179	0.5588	0.8378	0.9454	0.2835	0.5264	0.5789	0.7375	0.446	0.6959	0.8086	0.406	0.6524	0.5157	0.7281
*Eubacterium*,*Filifactor*	*r*	0.1826	0.2498	0.062	0.08542	0.08214	0.2473	0.3686	0.1809	0.2218	0.3314	-0.06755	0.0501	0.4034	-0.08002	-0.206
	*p*	0.2793	0.1359	0.7155	0.6152	0.6289	0.1401	0.0248*	0.2841	0.1871	0.0451*	0.6912	0.7684	0.0133*	0.6378	0.2213
AIH group		IL-1β	IL-4	IL-6	IL-7	IL-8	IL-12p70	IL-13	IL-17	G-CSF	IFN-γ	MCP-1	MIP-1β	TNF-α	IgA	Lysozyme
*Streptococcus*	*r*	0.1127	0.2063	0.3681	-0.03433	-0.1225	0.1989	0.2364	0.1357	0.1691	0.2505	-0.02451	-0.03556	0.1803	-0.03186	0.2328
	*p*	0.6666	0.427	0.146	0.8959	0.6394	0.4441	0.3611	0.6035	0.5164	0.3323	0.9256	0.8922	0.4887	0.9034	0.3685
*Porphyromonas*,*Prevotella*	*r*	0.3162	0.3794	0.04172	0.03801	0.2255	0.2824	-0.04116	0.3874	0.1789	0.4113	-0.2672	-0.206	0.3335	0.1838	-0.1863
	*p*	0.2163	0.1331	0.8737	0.8848	0.3842	0.2721	0.8754	0.1244	0.492	0.101	0.2999	0.4276	0.1908	0.48	0.4741
*Neisseria*	*r*	-0.2527	-0.03851	-0.4319	0.3054	-0.2198	0.008264	-0.1852	-0.01936	-0.3681	-0.06887	-0.1319	-0.1431	-0.06602	0.06593	-0.5495
	*p*	0.4043	0.8954	0.1383	0.3081	0.4703	0.9786	0.5448	0.9499	0.2159	0.8231	0.6676	0.6411	0.8303	0.8305	0.0518
*Veillonella*	*r*	0.5607	0.4027	0.5571	0.3311	0.5059	0.6126	0.3745	0.4602	0.4167	0.3659	0.2819	0.4267	0.2232	0.5613	0.3333
	*p*	0.0322*	0.109	0.0202*	0.1943	0.0456*	0.0089**	0.1386	0.063	0.0962	0.1487	0.2731	0.0876	0.3892	0.0191*	0.1911
*Eubacterium*	*r*	0.1154	-0.008253	0.08528	-0.2889	0.2747	0.1157	0.1672	-0.1853	0.2363	-0.03306	0.2033	0.4539	0.03026	-0.3022	-0.04396
	*p*	0.7097	0.9745	0.7818	0.3309	0.3637	0.7066	0.585	0.5444	0.4371	0.9146	0.5053	0.1192	0.9218	0.3156	0.8866
*Fusobacterium*	*r*	-0.3812	-0.166	0.1024	-0.02213	-0.3923	0.0277	0.07207	-0.242	-0.02762	-0.1994	-0.09945	0.2545	-0.177	0.0221	0.2265
	*p*	0.1928	0.5657	0.7382	0.9237	0.1795	0.9284	0.815	0.4257	0.9286	0.5136	0.7465	0.4014	0.5628	0.9429	0.4568
*Eubacterium*,*Parvimonas*	*r*	0.3236	0.366	0.08536	0.4239	0.4768	0.6215	-0.07854	0.323	0.1647	0.3202	0.1474	0.1346	0.2185	0.6877	-0.1214
	*p*	0.2774	0.2159	0.779	0.1482	0.1013	0.0265*	0.4564	0.277	0.5872	0.2816	0.6309	0.6612	0.4733	0.0094**	0.6929
*Eubacterium*,*Filifactor*	*r*	0.6332	0.5383	0.5862	0.5862	0.7108	0.5451	0.4481	0.6947	0.4898	0.5421	0.3046	0.1436	0.4067	0.6451	0.1434
	*p*	0.0238*	0.0617	0.0393*	0.0387*	0.009**	0.0571	0.1308	0.0113*	0.0925	0.0589	0.3108	0.6377	0.1686	0.0207*	0.641

Significant correlations after P-value adjustment are marked by

*P<0.05

**P<0.01

***P<0.0005.

### Correlation between the oral and gut microbiota in AILD patients

**Fig 3:**
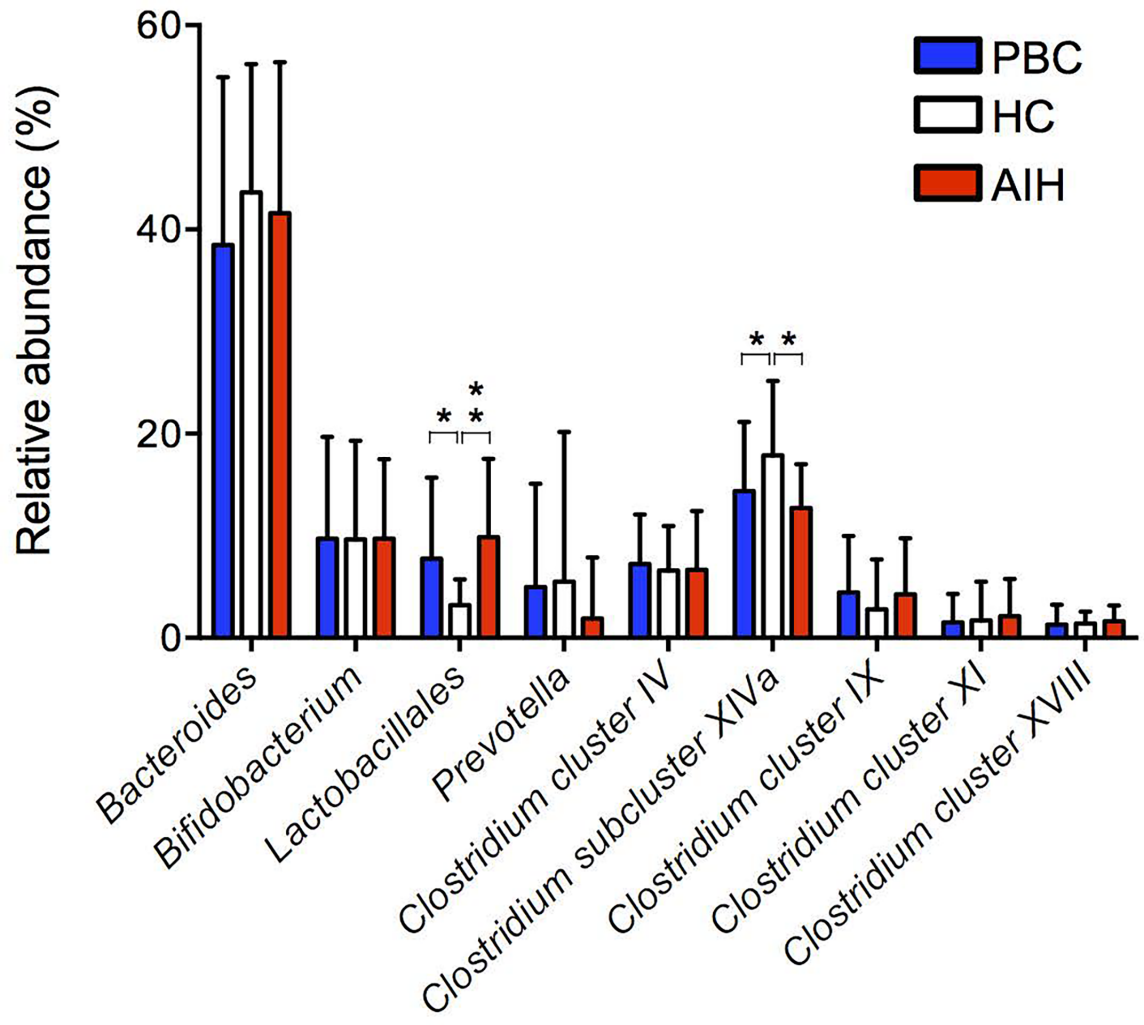
Mean genus or order abundance of gut microbiota in the PBC, AIH and HC groups. The plotted values are the mean abundance of the 8 abundant genera and 1 abundant order in each group. The results are expressed as the mean ± SD. Differences were compared using the Mann-Whitney U-test; *P<0.05, **P<0.01, ***P<0.0005.

Fig 3 shows the relative abundance of the bacterial composition at the genus or order level in fecal samples from subjects in the AIH, PBC and HC groups based on the T-RFLP profiles. The changes in the gut microbiota composition in AILD were characterized by an increase in the order *Lactobacillales* and by a decrease in the genus *Clostridium subcluster XIVa*. We next examined for correlations between the relative abundance of bacterial composition in salivary samples and that in fecal samples form patients with AILD. The results are shown in Table 3. The relative abundance of *Lactobacillales* in feces positively correlated with the relative abundance of *Veillonella* in saliva from patients with AIH, whereas the relative abundance of *Bifidobacterium* in feces negatively correlated with the relative abundance of *Veillonella* in saliva from patients with PBC. Moreover, the relative abundance of *Clostridium subcluster XIVa* in feces positively correlated with the relative abundance of *Neisseria* and negatively correlated with the relative abundance of *Eubacterium* in saliva from patients with AIH. By contrast, the abundance of *Streptococcus* in saliva positively correlated with the abundance of *Clostridium cluster XVIII* and negatively correlated with the relative abundance of *Bifidobacterium* in feces from patients with AIH.

**Table 3:** Correlation between oral microbiota and gut microbiota in AILD patients.

PBC group		*Bacteroides*	*Bifidobacterium*	*Lactobacillales*	*Prevotella*	*Clostridium* *cluster IV*	*Clostridium* *subcluster XIVa*	*Clostridium* *cluster IX*	*Clostridium* *cluster XI*	*Clostridium* *cluster XVIII*
*Streptococcus*	*r*	-0.2562	-0.06162	0.07458	0.2159	0.03826	-0.009416	0.1714	0.01261	0.05467
	*p*	0.0577	0.3547	0.3259	0.0934	0.4086	0.4773	0.1484	0.4696	0.3705
*Porphyromonas*,*Prevotella*	*r*	0.2184	0.05726	-0.0307	-0.1827	-0.01245	0.04303	-0.1205	-0.03221	-0.08703
	*p*	0.1817	0.7292	0.8528	0.2656	0.94	0.7948	0.4648	0.8457	0.5983
*Neisseria*	*r*	0.2775	-0.1193	-0.1861	0.08657	-0.03766	0.07907	-0.1543	0.01428	-0.08077
	*p*	0.0872	0.4695	0.2566	0.6002	0.82	0.6323	0.3482	0.9312	0.625
*Veillonella*	*r*	0.0327	-0.3697	0.2091	-0.04718	0.171	0.08413	0.05185	0.002919	-0.1108
	*p*	0.4217	0.0103*	0.1007	0.3878	0.149	0.3053	0.377	0.493	0.251
*Eubacterium*	*r*	0.2161	0.2295	0.1224	-0.198	-0.1488	-0.04667	0.1495	-0.1337	-0.2556
	*p*	0.1863	0.1598	0.4579	0.2269	0.366	0.7778	0.3638	0.417	0.1164
*Fusobacterium*	*r*	-0.07157	0.1242	-0.2783	-0.06005	0.2005	0.2557	0.1559	0.2495	0.132
	*p*	0.665	0.4514	0.0862	0.7165	0.2209	0.1161	0.3431	0.1256	0.423
*Eubacterium*,*Parvimonas*	*r*	0.1522	0.05446	0.04538	-0.1733	-0.141	0.03482	-0.04523	0.1619	-0.1403
	*p*	0.3551	0.742	0.7838	0.2914	0.3919	0.8333	0.7845	0.3249	0.3943
*Eubacterium*,*Filifactor*	*r*	0.0475	-0.0284	-0.2434	0.04796	0.1345	0.05774	0.09116	0.1054	0.03062
	*p*	0.774	0.8638	0.1354	0.7718	0.4144	0.727	0.581	0.5231	0.8532
AIH group		*Bacteroides*	*Bifidobacterium*	*Lactobacillales*	*Prevotella*	*Clostridium* *cluster IV*	*Clostridium* *subcluster XIVa*	*Clostridium* *cluster IX*	*Clostridium* *cluster XI*	*Clostridium* *cluster XVIII*
*Streptococcus*	*r*	0.1446	-0.5441	0.2868	NA	-0.3372	-0.2108	0.1215	-0.03815	0.5108
	*p*	0.5798	0.0239*	0.2644	NA	0.1856	0.4167	0.6423	0.8844	0.0361*
*Porphyromonas*,*Prevotella*	*r*	0.1348	-0.2353	-0.07598	NA	0.2183	0.3309	-0.1387	0.3217	0.08143
	*p*	0.606	0.3633	0.7719	NA	0.4	0.1945	0.5956	0.208	0.756
*Neisseria*	*r*	0.1814	0.2304	-0.3701	NA	0.4513	0.5221	-0.06135	0.2314	-0.2751
	*p*	0.486	0.3737	0.1437	NA	0.069	0.0316*	0.8151	0.3715	0.2851
*Veillonella*	*r*	-0.4706	-0.1593	0.777	NA	-0.1018	-0.1716	-0.07117	0.4221	0.29
	*p*	0.0566	0.5414	0.0002***	NA	0.6975	0.5103	0.7861	0.0914	0.2589
*Eubacterium*	*r*	0.1544	0.2279	-0.2108	NA	-0.2943	-0.5172	-0.2994	-0.09155	-0.05305
	*p*	0.554	0.3789	0.4167	NA	0.2515	0.0335*	0.243	0.7268	0.8397
*Fusobacterium*	*r*	-0.407	-0.2308	0.2655	NA	0.0509	0.01241	-0.004969	0.1365	-0.1274
	*p*	0.105	0.3728	0.303	NA	0.8462	0.9623	0.9849	0.6015	0.626
*Eubacterium*,*Parvimonas*	*r*	-0.406	-0.1698	0.02388	NA	0.3107	0.3397	-0.08901	0.424	-0.07882
	*p*	0.1058	0.5146	0.9275	NA	0.2248	0.1822	0.7341	0.0898	0.7636
*Eubacterium*,*Filifactor*	*r*	-0.07962	-0.2574	0.6024	NA	-0.1075	-0.146	0.182	-0.03993	0.09352
	*p*	0.7613	0.3185	0.0105*	NA	0.6812	0.5762	0.4844	0.8791	0.7211

Significant correlations after P-value adjustment are marked by

*P<0.05

**P<0.01

***P<0.0005. NA, not available.

### Associations between clinical variables and the oral microbiota

We investigated the effects of subphenotypes on the oral microbiota in AILD patients (S2 Fig). We examined whether sex bias in AILD patients was associated with the oral microbiome. The relative abundance of the bacterial composition at the genus level in salivary samples was not significantly related to sex in this study (S2A and S2B Fig). The patients were divided into advanced and non-advanced stages based on the Scheuer system and fibrosis. There was no significant difference between the two stages in the relative abundance of associated PBC and AIH taxa (S2C and S2D Fig). There was a significantly higher frequency of genus *Neisseria* (OTU496) in salivary samples obtained from AIH patients with abnormal liver function than in those obtained from AIH patients with normal liver function, whereas there was a significantly lower frequency of genus *Neisseria* in PSL-using AIH patients than in non-PSL-using AIH patients. Moreover, there was a significantly higher frequency of genus *Streptococcus* (OUT556, 563) in UDCA 600–900 (mg/day) users than in UDCA 0–300 (mg/day) users among AIH patients. There was no significant difference between PBC patients who were treated with or without medications such as UDCA and bezafibrate (S2E–S2J Fig).

Moreover, we next investigated the effects of subphenotypes on the gut microbiota in AILD patients (S3 Fig). There was a significantly lower frequency of the genus *Clostridium cluster IX* in fecal samples obtained from female patients than in fecal samples obtained from male PBC patients (S3A Fig). Moreover, there was a significantly lower frequency of the genera *Clostridium cluster IV* and *Clostridium subcluster XIVa* in fecal samples obtained from PBC patients with abnormal liver function than in samples obtained from PBC patients with normal liver function (S3E Fig). There were no significant differences in the relative abundance of bacterial composition with respect to sex, disease stage, UDCA and PSL use among AIH patients.

### Principal component analysis (PCA)

**Fig 4:**
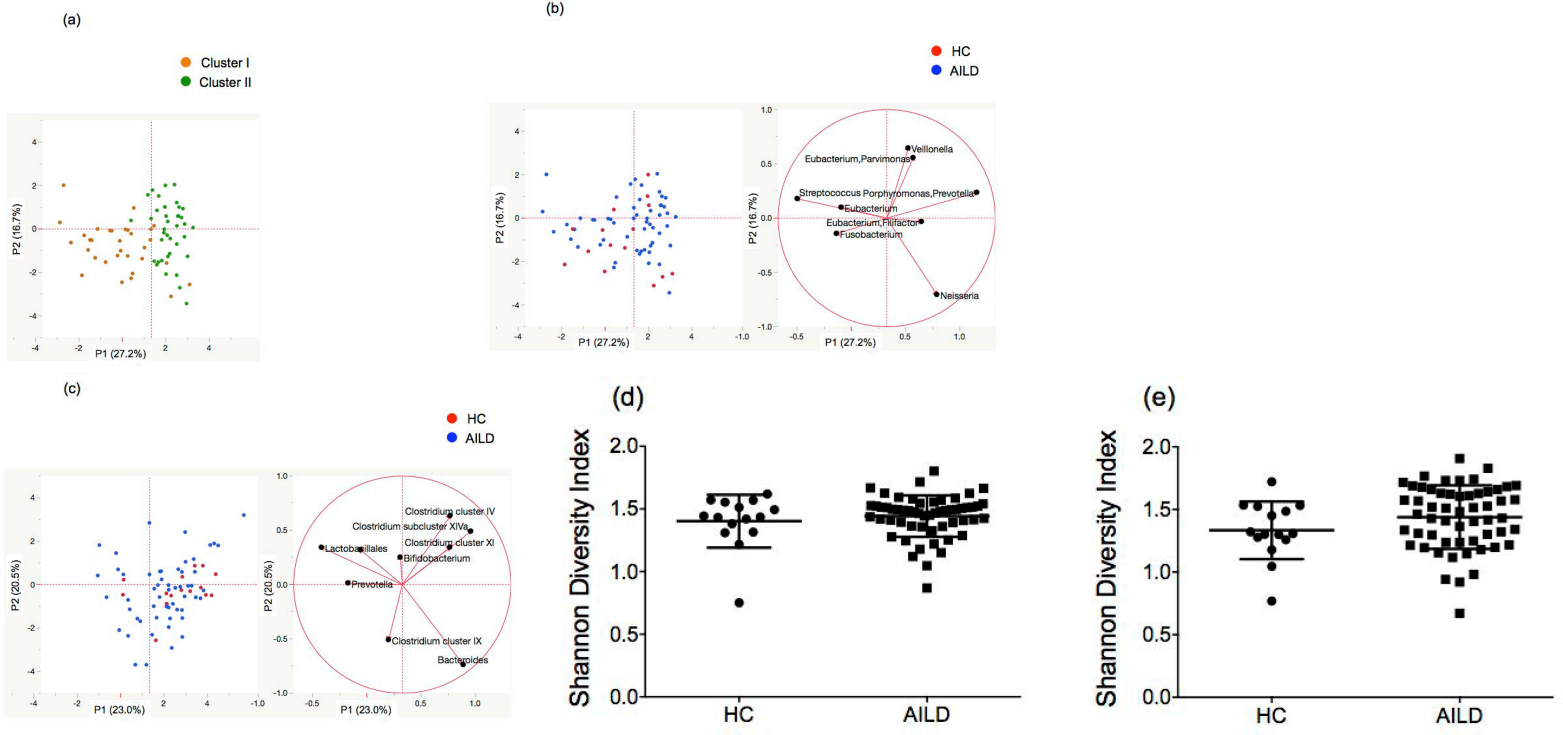
Principal component analysis (PCA) of the oral and gut microbiota among the 71 T-RFLP profiles. (a) The T-RFLP profiles were classified into two clusters by hierarchical cluster analysis (orange circle: Cluster I, green circle: Cluster II). (b) Principal component analysis of the oral microbiota in the AILD (blue circle) and HC groups (red circle). (c) Principal component analysis of the gut microbiota in the AILD (blue circle) and HC groups (red circle). (d) Index (Shannon, y-axis) of genera diversity in oral microbiota, (e) Index (Shannon, y-axis) of genera diversity in gut microbiota.

We created the distribution map based on the PCA to visualize the difference in T-RFLP profiles of oral microbiota between the AILD and HC groups and found that the first and second principal components explained 43.9% of the variance (Fig 4A and 4B). Subjects of Cluster I were localized in the left part of this map, and subjects of Cluster II were localized in the right part (Fig 4A). The PCA showed a relatively weak clustering of the oral microbiota between the AILD and HC groups (Fig 4B). The AILD-related genera included *Veillonella*, while *Fusobacterium* was more related to the HC samples. The *Streptococcus*, *Eubacterium* and *Neisseria* genera were approximately between the AILD and HC groups. Fig 4C presents the PCA of the gut microbiota in the AILD and HC groups. The two components explained 43.5% of the variance. Gut microbiota showed more clustering in the AILD group than in the HC group. At the genera level, we found no significant difference between the groups in regard to the Shannon Diversity index of oral microbiota (Fig 4D, *P* = 0.594) and gut microbiota (Fig 4E, *P* = 0.1325).

### Univariate and multivariate analyses of microbiota associated with AILD patients

We investigated the association between microbial flora and AILD patients using univariate and multivariate analyses (Table 4). Univariate analysis showed significant associations between AILD patients and the increased relative abundance of *Veillonella* in oral microbiota, the increased relative abundance of *Lactobacillales* and the decreased relative abundance of *Clostridium subcluster XIVa* in gut microbiota. The subsequent multivariate analysis showed that the genus *Veillonella* in oral microbiota (odds ratio [OR]: 1.49, 95% confidence interval [CI]: 1.14–1.94, P = 0.003) was independently associated with AILD patients.

**Table 4:** Univariate and multivariate analyses of microbiota associated with AILD patients.

AILD vs. HC	Univariate		Multivariate	
	OR (95% CI)	P	OR (95% CI)	P
**Oral microbiota**				
*Streptococcus*	0.96 (0.92–1.01)	0.131		
*Porphyromonas*,*Prevotella*	1.04 (0.97–1.12)	0.299		
*Neisseria*	0.98 (0.92–1.05)	0.597		
*Veillonella*	1.50 (1.17–1.93)	0.001	1.49 (1.14–1.94)	0.003
*Eubacterium*	1.65 (1.00–2.74)	0.052		
*Fusobacterium*	0.71 (0.49–1.03)	0.074		
*Eubacterium*,*Parvimonas*	0.64 (0.25–1.64)	0.351		
*Eubacterium*,*Filifactor*	0.61 (0.34–1.09)	0.096		
**Gut microbiota**				
*Bacteroides*	0.98 (0.94–1.02)	0.344		
*Bifidobacterium*	1.00 (0.94–1.06)	0.976		
*Lactobacillales*	1.21 (1.02–1.43)	0.03	1.15 (0.92–1.43)	0.223
*Prevotella*	0.99 (0.94–1.04)	0.624		
*Clostridium cluster IV*	1.02 (0.91–1.15)	0.741		
*Clostridium subcluster XIVa*	0.92 (0.84–1.00)	0.044	0.92 (0.84–1.02)	0.102
*Clostridium cluster IX*	1.07 (0.93–1.23)	0.322		
*Clostridium cluster XI*	1.00 (0.83–1.19)	0.983		
*Clostridium cluster XVIII*	1.00 (0.71–1.40)	0.99		

OR, odds ratio; CI, confidence interval

## Discussion

Until recently, there have been almost no studies exhaustively examining the oral microbiota at the genus level in subjects with AILD.

In this study, we used T-RFLP analysis and found that the oral microbiota T-RFLP profile of subjects with AILD was significantly different from that of HCs. Our data indicated a significant increase in the genus *Veillonella* in the salivary microbiota of AILD patients; its relative abundance was almost equivalent to the reduced abundance of *Streptococcus*, which is most abundant in healthy salivary microbiota. The genus *Veillonella* is an anaerobic gram-negative coccus that is part of the normal flora of the human mouth and gastrointestinal tract [32]. The main habitats of *Veillonella* are the tongue, buccal mucosa, and saliva [33]. The *Veillonella* genus has recently been associated with primary sclerosing cholangitis and PBC [19, 34]. *Veillonella* is associated with poor oral health, which causes many human oral infectious diseases, such as periodontitis [35]. *Veillonella* produces a large amount of lipopolysaccharide to induce cytokine secretion [36]. In this study, our data indicated that the abundance of *Veillonella* positively correlated with the levels of pro-inflammatory cytokines, such as IL-1β, IL-6, IL-8, and IL-12p70 in the saliva of patients with AIH. These data suggest that the increase in *Veillonella* is clearly related to abnormal physiologies in AILD patients. The subjects could be divided into two groups based on cluster classification using the T-RFLP profiles of their saliva. Approximately 61% of subjects with AILD were categorized into the Cluster II microbiota, while approximately 67% of the HCs were categorized into the Cluster I microbiota. The characteristics of Cluster II, comprising most subjects with AILD, included a lower frequency of genera *Streptococcus* and *Fusobacterium* and a higher frequency of genus *Veillonella*. A previous study showed that the genus *Veillonella* was significantly higher in the salivary microbiota of inflammatory bowel disease (IBD) patients than in that of HCs, while the genus *Streptococcus* was significantly lower in the salivary microbiota of IBD patients than in that of HCs [12]. Moreover, the relative abundance of *Streptococcus* negatively correlated with the levels of IL-1β and IL-8, while that of *Veillonella* tended to positively correlate with the levels of cytokines and secretary IgA in the saliva of IBD patients. In this study, the relative abundance of *Streptococcus* negatively correlated with the levels of cytokines, such as L-1β and IL-8, while the relative abundance of *Veillonella* positively correlated with the salivary IgA level of patients with PBC. Multivariate analysis showed that the increased relative abundance of *Veillonella* in oral microbiota was independently associated with AILD patients.

Recent studies have reported that PPIs affect both the gut and oral microbiota [37, 38]. After administration of PPIs for 4 weeks, alterations of the microbiota in the oral carriage microbiome along with bacterial overgrowth (*Streptococcus*) and decreases in distinct bacterial species (*Neisseria*, *Veillonella*) were observed in healthy volunteers. In this study, our estimates revealed that the oral microbiota of PPI users was similar to that of non-PPI users among patients with AILD.

Reduced salivation is a major clinical feature of most cases of Sjögren’s syndrome. Reduced saliva may lead to changes in the salivary microbiota. A recent report indicated that the genera *Streptococcus* and *Veillonella* were significantly higher in patients with Sjögren’s syndrome than in controls [39]. In this study, Sjögren’s syndrome was associated with 1 case of PBC (PBC36) and 2 cases of AIH (AIH4, AIH10). Indeed, the relative abundance of *Veillonella* was high in AILD patients with Sjögren’s syndrome, but even after excluding those patients, the relative abundance of *Veillonella* was significantly higher in AILD patients than in HCs (PBC, 8.4% vs 4.6%, *p*<0.0005, AIH, 9.8% vs 4.6%, *p*<0.001).

Saliva contains a variety of components such as cytokines, immunoglobulins, and antimicrobial proteins involved in host defense mechanisms for maintaining oral and systemic health [40]. Alterations in the salivary microbiota of cirrhosis patients with hepatic encephalopathy suggest the occurrence of an inflammatory immune response in the oral cavity of cirrhosis patients as intestinal inflammation is associated with the gut microbiota of cirrhosis [14]. In this study, the levels of many pro-inflammatory cytokines, such as IL-1β, IFN-γ, and secretory IgA, were significantly higher in both AIH and PBC patients than in HCs. A previous study reported that IL-6 and IFN-γ levels were significantly increased in the saliva of PBC patients. Moreover, the IL-6 and IFN-γ levels in the saliva of PBC patients are positively associated with those in the sera of those patients [41]. Similarly, elevated levels of salivary IL-1β, IL-6, and secretory IgA in cirrhosis patients have also been reported [14]. However, it is unknown whether the inflammatory state in the oral cavity of AILD patients is the cause or a consequence of imbalances in the salivary microbiota and whether the oral cavity or the gut immune response is more responsible for the observed dysbiosis of the oral microbiota. In this study, the changes in gut microbiota composition in AILD were characterized by an increase in the order *Lactobacillales* and by a decrease in the genus *Clostridium subcluster XIVa*. Previous reports have revealed that *Lactobacillus* species were more prevalent and that *Clostridia* was less frequent in the gut microbiota of patients with Behcet’s disease than in HCs [42]. *Lactobacillus* species had relatively large effect sizes in Behcet’s disease microbiota, which is concordant with the inductive effect of *Lactobacillus* on systemic inflammation. Animal studies using germ-free mice reported that some bacterial species separately promoted arthritis by activating Th17 cells [43, 44]. Indeed, oral intake of *Lactobacillus* rapidly induced arthritis in genetically modified germ-free mice [43]. *Clostridium* species have been suggested to activate regulatory T cells (Treg) and then modulate mucosal immune system through the production of short chain fatty acids [45]. *Lactobacillus* are major lactate-producing and pH-regulating bacteria with the consumption of hexose sugars [46]. In contrast to the lactate production, several genera of the order *Clostridiales* can utilize lactate and produce butyrate or propionate [47, 48].

Interestingly, our study suggested that while the relative abundance of *Lactobacillales* in feces positively correlated with the relative abundance of *Veillonella* in saliva from patients with AIH, the relative abundance of *Bifidobacterium* in feces negatively correlated with the relative abundance of *Veillonella* in saliva from patients with PBC. Moreover, the relative abundance of *Clostridium subcluster XIVa* in feces positively correlated with the relative abundance of *Neisseria* and negatively correlated with the relative abundance of *Eubacterium* in saliva from patients with AIH. Dysbiosis of the oral microbiota reflects changes in the gut microbiota in patients with AILD. Recent studies have shown that *Clostridia clusters XIVa*, *IV* derived from human feces have the potential to induce Foxp3+ Tregs and are able to suppress inflammatory conditions such as colitis, experimental autoimmune encephalomyelitis, and multiple sclerosis [49–51]. AIH is predominately associated with Th1 responses and the decreased function and number of Tregs [52, 53]. Dysbiosis of the oral microbiota is directly and/or indirectly related to the gut microbiota and may be correlated with disease onset.

We examined the effects of subphenotypes on the oral microbiota in AILD patients. There were no significant differences in the relative abundance of the oral microbiota with respect to sex and disease stage among AILD patients. A previous study revealed that microbial dysbiosis in PBC was partially relieved after UDCA treatment [19]. In this study, there was no significant difference between PBC patients treated with and those treated without medications such as UDCA and bezafibrate; most PBC patients were treated with UDCA at sample collection. There was a significantly higher frequency of the genus *Neisseria* in salivary samples obtained from AIH patients with abnormal liver function than in those obtained from AIH patients with normal liver function, but there was a significantly lower frequency of the genus *Neisseria* in PSL users than in non-PSL users among AIH patients. Thus, *Neisseria* may be involved in the exacerbation of AIH.

Our study has some limitations. First, the sample population was relatively small. Second, we did not evaluate changes in the salivary and fecal microbiota that might have occurred due to treatment in AILD patients.

## Conclusions

This may be the first report demonstrating dysbiosis of the oral microbiota in patients with AIH or PBC. These findings suggest that the oral microbiota may play different roles in the pathophysiology of AIH and PBC. Further studies of the establishment and modification of the oral microbiota structure may contribute to the development of a therapeutic strategy for patients with AILD.

## Supporting information

S1 Table Levels of non-significant cytokines in the saliva of HCs and patients with AIH or PBC. (DOCX)

S1 Fig Bacterial composition at the genus or order level in salivary and fecal microbiota samples obtained from proton pump inhibitor (PPI) users and non-users. The salivary microbiota of PPI users was not significantly different from that of non-PPI users among patients with AIH or PBC. Mean genus abundance in the (a) PBC and (b) AIH groups. The plotted values are the mean abundance of the 8 abundant genera in each group. The fecal microbiota of PPI users was not significantly different from that of non-PPI users among patients with PBC. There was a significantly lower frequency of the genus *Bifidobacterium* (OTU124) in fecal samples obtained from PPI users than in those obtained from non-PPI users among AIH patients. The mean genus or order abundance in the (c) PBC and (d) AIH groups. The plotted values are the mean abundance of the 8 abundant genera and 1 abundant order in each group. The open and filled bars represent samples obtained from PPI users and non-PPI users. The results are expressed as the mean ± SD. Differences were compared using the Mann-Whitney U-test; *P<0.05.
(PPTX)

S2 Fig Associations between clinical variables and oral microbiota. The mean genus abundance in (a) male and female patients with PBC; (b) male and female patients with AIH; (c) Scheuer 1–2 and Scheuer 3–4 patients with PBC; (d) F0-2 and F3-4 patients with AIH; (e) PBC patients with normal liver function and abnormal liver function; (f) AIH patients with normal liver function and abnormal liver function; (g) UDCA 0–300 mg/day users and UDCA 600–900 mg/day users among patients with PBC; (h) UDCA 0–300 mg/day users and UDCA 600–900 mg/day users among patients with AIH; (i) bezafibrate (BF) users and non-BF users among patients with PBC; (j) prednisolone (PSL) users and non-PSL users among patients with AIH. The results are expressed as the mean ± SD. Differences were compared using the Mann-Whitney U-test; *P<0.05.
(PPTX)

S3 Fig Associations between clinical variables and gut microbiota. The mean genus abundance in (a) male and female patients with PBC; (b) male and female patients with AIH; (c) Scheuer 1–2 and Scheuer 3–4 patients with PBC; (d) F0-2 and F3-4 patients with AIH; (e) PBC patients with normal liver function and abnormal liver function; (f) AIH patients with normal liver function and abnormal liver function; (g) UDCA 0–300 mg/day users and UDCA 600–900 mg/day users among patients with PBC; (h) UDCA 0–300 mg/day users and UDCA 600–900 mg/day users among patients with AIH; (i) bezafibrate (BF) users and non-BF users among patients with PBC; (j) prednisolone (PSL) users and non-PSL users among patients with AIH. The results are expressed as the mean ± SD. Differences were compared using the Mann-Whitney U-test; *P<0.05.
(PPTX)
