# Supplementary material for: Dysbiosis of oral microbiota and its association with salivary immunological biomarkers in autoimmune liver disease
Source: PLoS One. 2018 Jul 3;13(7):e0198757. doi: 10.1371/journal.pone.0198757 (PMC6029758; doi:10.1371/journal.pone.0198757)
Supplement: S1 Table — (DOCX) [file pone.0198757.s001.docx]

**Supporting Table 1.** Levels of non-significant cytokines in the saliva of HCs and patients with AILD or PBC

| Cytokine (pg/ml) | PBC | | AIH | | HC | |
| --- | --- | --- | --- | --- | --- | --- |
|  | Detection  (%) | Mean  ± sd | Detection (%) | Mean ± sd | Detection (%) | Mean ± sd |
| IL-2 | 5 (12.8%) | 1.4±6.2 | 1 (5.9%) | 0.2±0.8 | 1 (6.7%) | 0.3±1.3 |
| IL-4 | 31 (79.5%) | 1.8±4.0 | 15 (88.2%) | 1.5±1.7 | 8 (53.3%) | 0.8±2.0 |
| IL-5 | 5 (12.8%) | 0.9±3.6 | 1 (5.9%) | 0.4±1.5 | 2 (13.3%) | 0.4±1.5 |
| IL-6 | 36 (92.3%) | 13.9±27.6 | 17 (100%) | 17.8±41.0 | 11 (73.3%) | 6.3±9.4 |
| IL-7 | 39 (100%) | 8.8±11.2 | 17 (100%) | 5.5±4.9 | 15 (100%) | 3.4±4.2 |
| IL-10 | 3 (7.7%) | 2.3±10.3 | 1 (5.9%) | 0.8±3.1 | 1 (6.7%) | 1.2±4.6 |
| IL-12p70 | 34 (87.2%) | 5.8±20.5 | 15 (88.2%) | 2.8±6.4 | 11 (73.3%) | 4.0±9.5 |
| IL-13 | 13 (33.3%) | 2.0±7.9 | 8 (47.1%) | 0.7±2.4 | 6 (40.0%) | 1.2±3.5 |
| IL-17 | 23 (60.0%) | 8.5±19.2 | 13 (76.5%) | 5.5±7.6 | 4 (26.7%) | 2.2±5.6 |
| G-CSF | 39 (100%) | 72.2±94.3 | 17 (100%) | 178.7±353.9 | 15 (100%) | 35.5±57.9 |
| GM-CSF | 1 (2.6%) | 0.2±1.1 | 0 (0%) | 0.0±0.0 | 0 (0%) | 0.0±0.0 |
| MCP-1 | 38 (97.4%) | 298.5±271.4 | 16 (94.1%) | 316.3±239.5 | 14 (93.3%) | 187.9±195.3 |
| Lysozyme | 39 (100%) | 9.5±7.3x10^6^ | 17 (100%) | 7.7±7.8x10^6^ | 13 (86.7%) | 7.5±7.9x10^6^ |
