# Supplementary figures and images for: Dysbiosis of oral microbiota and its association with salivary immunological biomarkers in autoimmune liver disease
Source: PLoS One. 2018 Jul 3;13(7):e0198757. doi: 10.1371/journal.pone.0198757 (PMC6029758; doi:10.1371/journal.pone.0198757)

## Slide 1
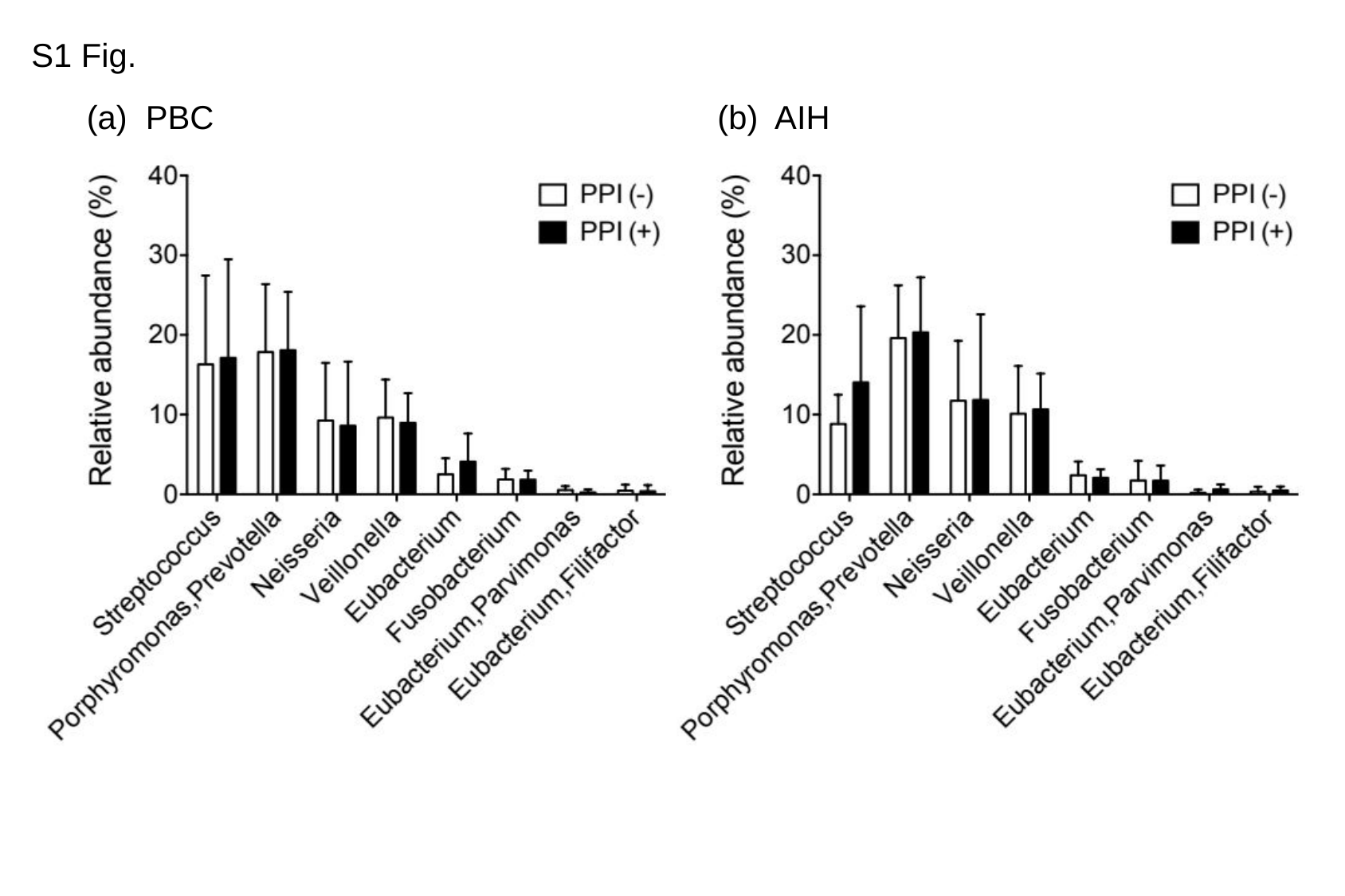

S1 Fig.
(a) PBC
(b) AIH

## Slide 2
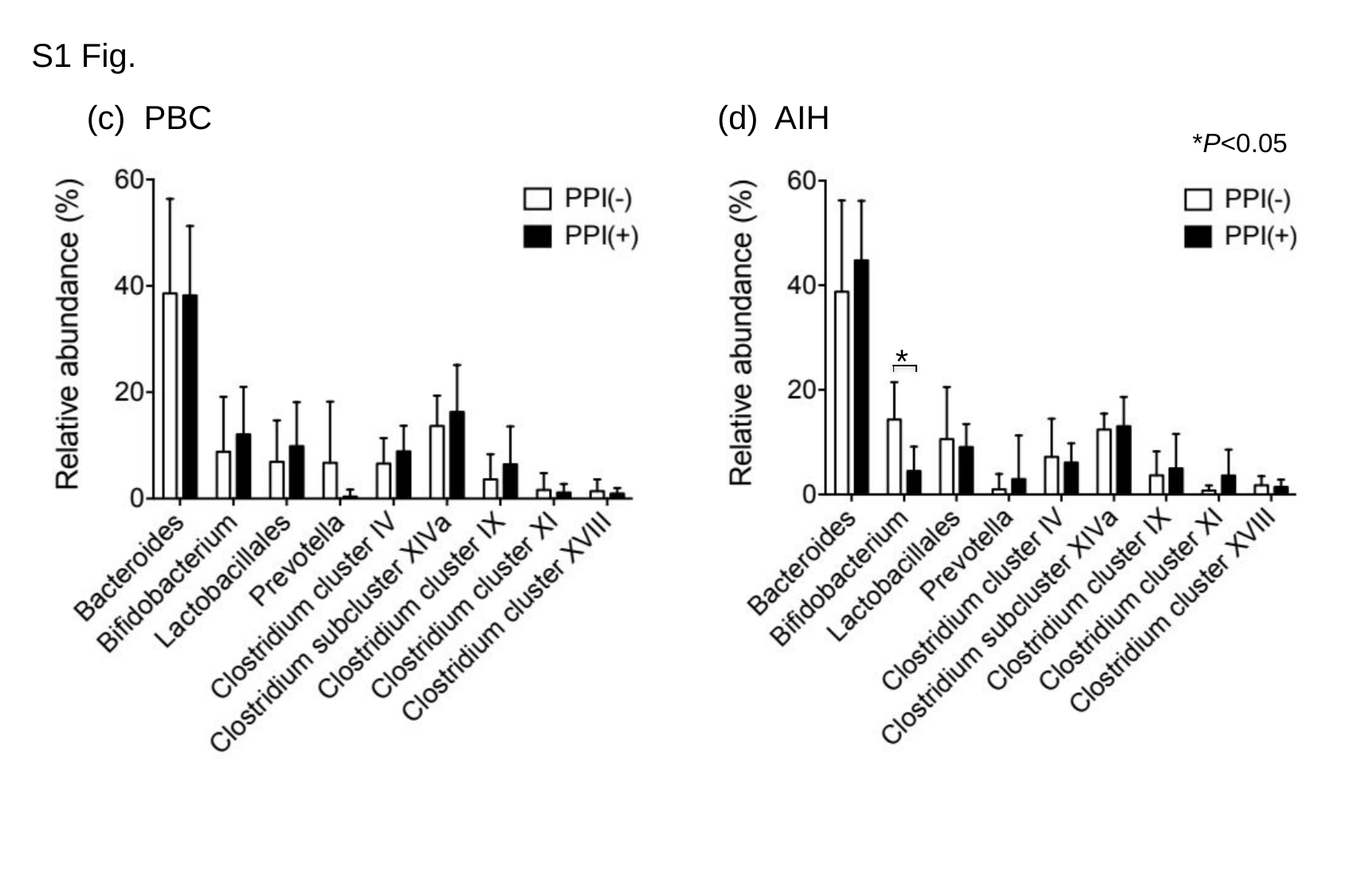

S1 Fig.
(c) PBC
(d) AIH
 *P<0.05
*

Supplement: S1 Fig — The salivary microbiota of PPI users was not significantly different from that of non-PPI users among patients with AIH or PBC. Mean genus abundance in the (a) PBC and (b) AIH groups. The plotted values are the mean abundance of the 8 abundant genera in each group. The fecal microbiota of PPI users was not significantly different from that of non-PPI users among patients with PBC. There was a significantly lower frequency of the genus Bifidobacterium (OTU124) in fecal samples obtained from PPI users than in those obtained from non-PPI users among AIH patients. The mean genus or order abundance in the (c) PBC and (d) AIH groups. The plotted values are the mean abundance of the 8 abundant genera and 1 abundant order in each group. The open and filled bars represent samples obtained from PPI users and non-PPI users. The results are expressed as the mean ± SD. Differences were compared using the Mann-Whitney U-test; *P<0.05. (PPTX) [file pone.0198757.s002.pptx]
