# Supplementary material for: Dysbiosis of oral microbiota and its association with salivary immunological biomarkers in autoimmune liver disease
Source: PLoS One. 2018 Jul 3;13(7):e0198757. doi: 10.1371/journal.pone.0198757 (PMC6029758; doi:10.1371/journal.pone.0198757)
Supplement: S2 Fig — The mean genus abundance in (a) male and female patients with PBC; (b) male and female patients with AIH; (c) Scheuer 1–2 and Scheuer 3–4 patients with PBC; (d) F0-2 and F3-4 patients with AIH; (e) PBC patients with normal liver function and abnormal liver function; (f) AIH patients with normal liver function and abnormal liver function; (g) UDCA 0–300 mg/day users and UDCA 600–900 mg/day users among patients with PBC; (h) UDCA 0–300 mg/day users and UDCA 600–900 mg/day users among patients with AIH; (i) bezafibrate (BF) users and non-BF users among patients with PBC; (j) prednisolone (PSL) users and non-PSL users among patients with AIH. The results are expressed as the mean ± SD. Differences were compared using the Mann-Whitney U-test; *P<0.05. (PPTX) [file pone.0198757.s003.pptx]

## Slide 1
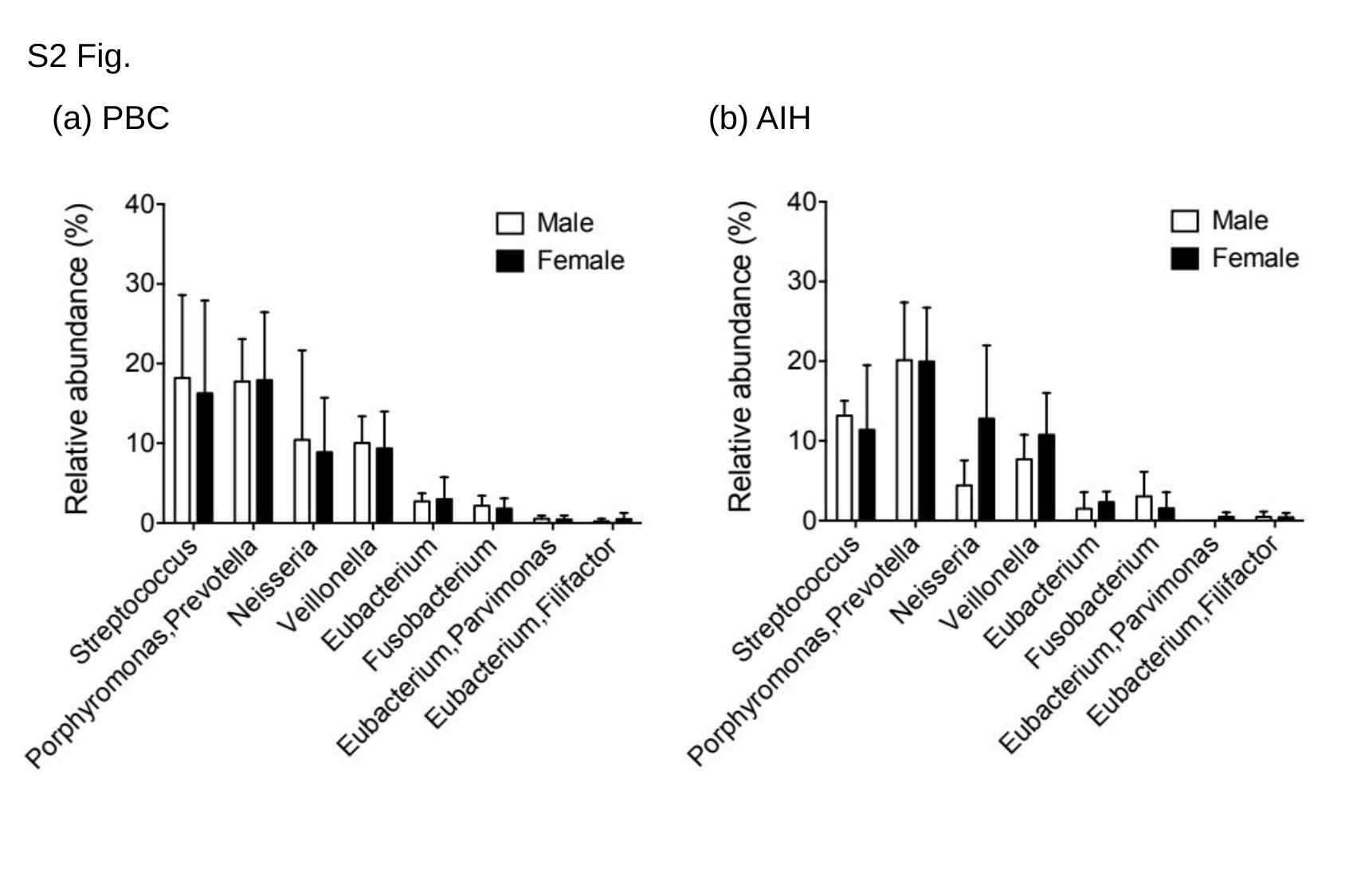

S2 Fig.
(a) PBC
(b) AIH

## Slide 2
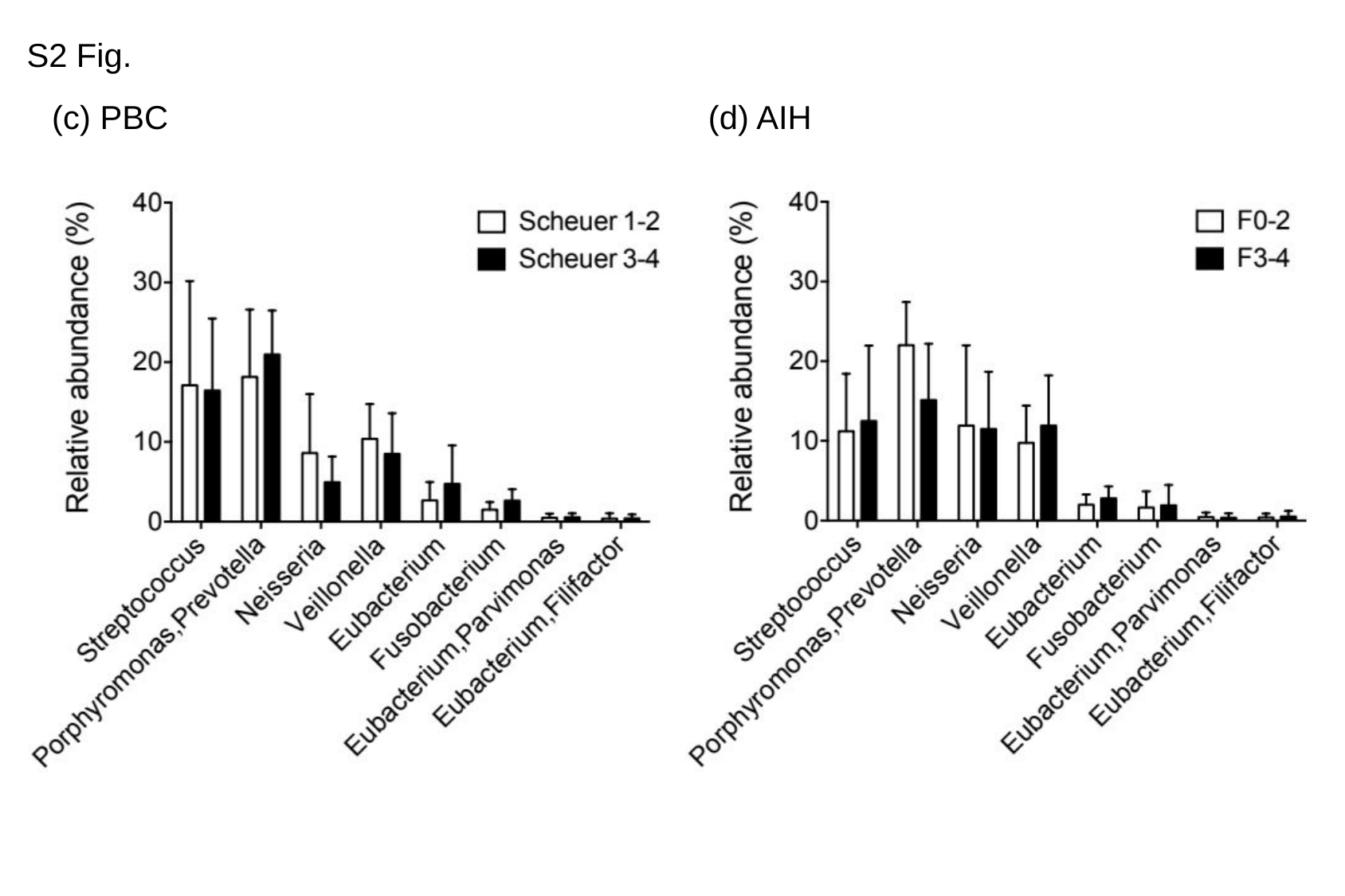

S2 Fig.
(c) PBC
(d) AIH

## Slide 3
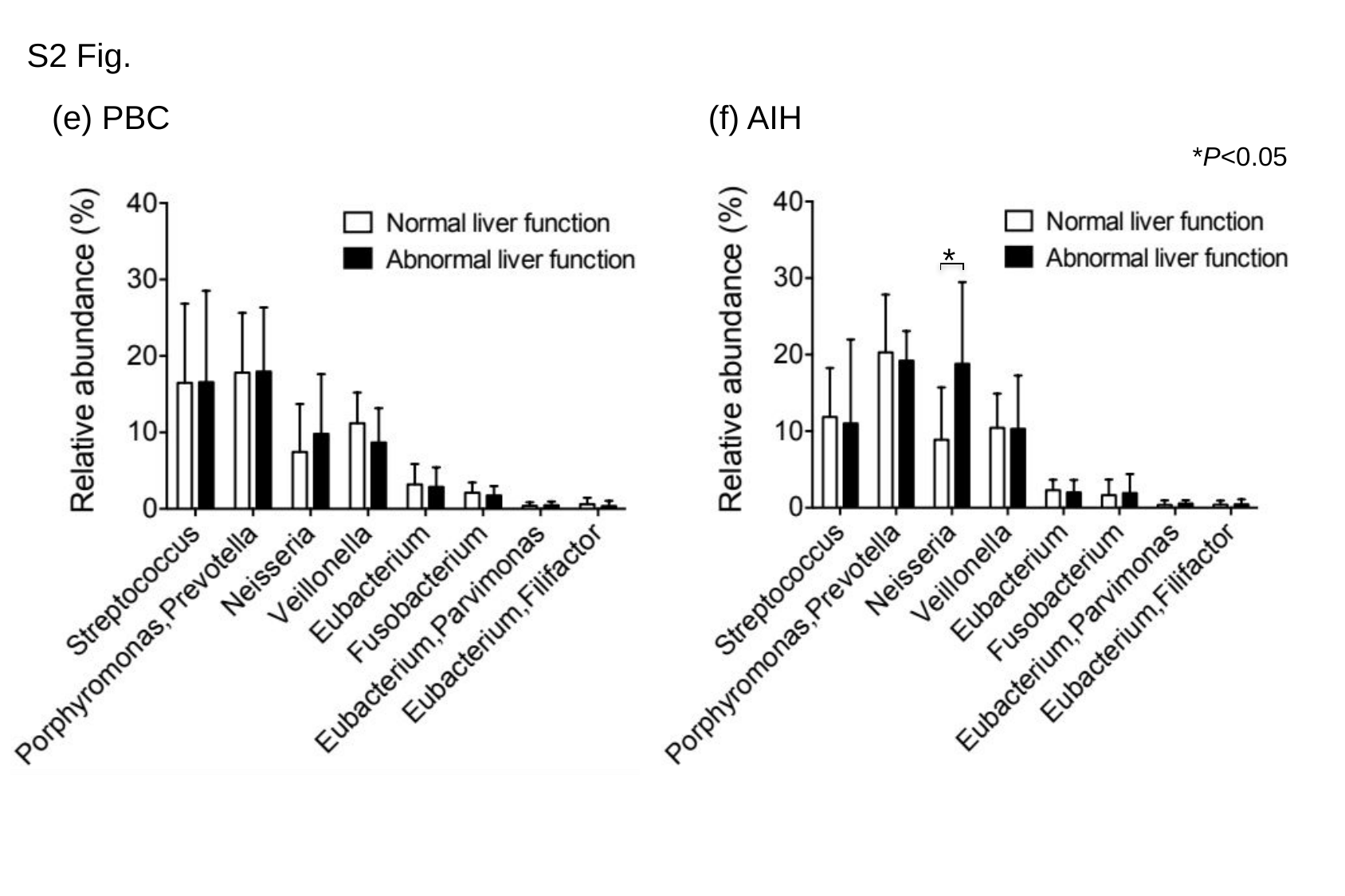

S2 Fig.
(e) PBC
(f) AIH
 *P<0.05
*

## Slide 4
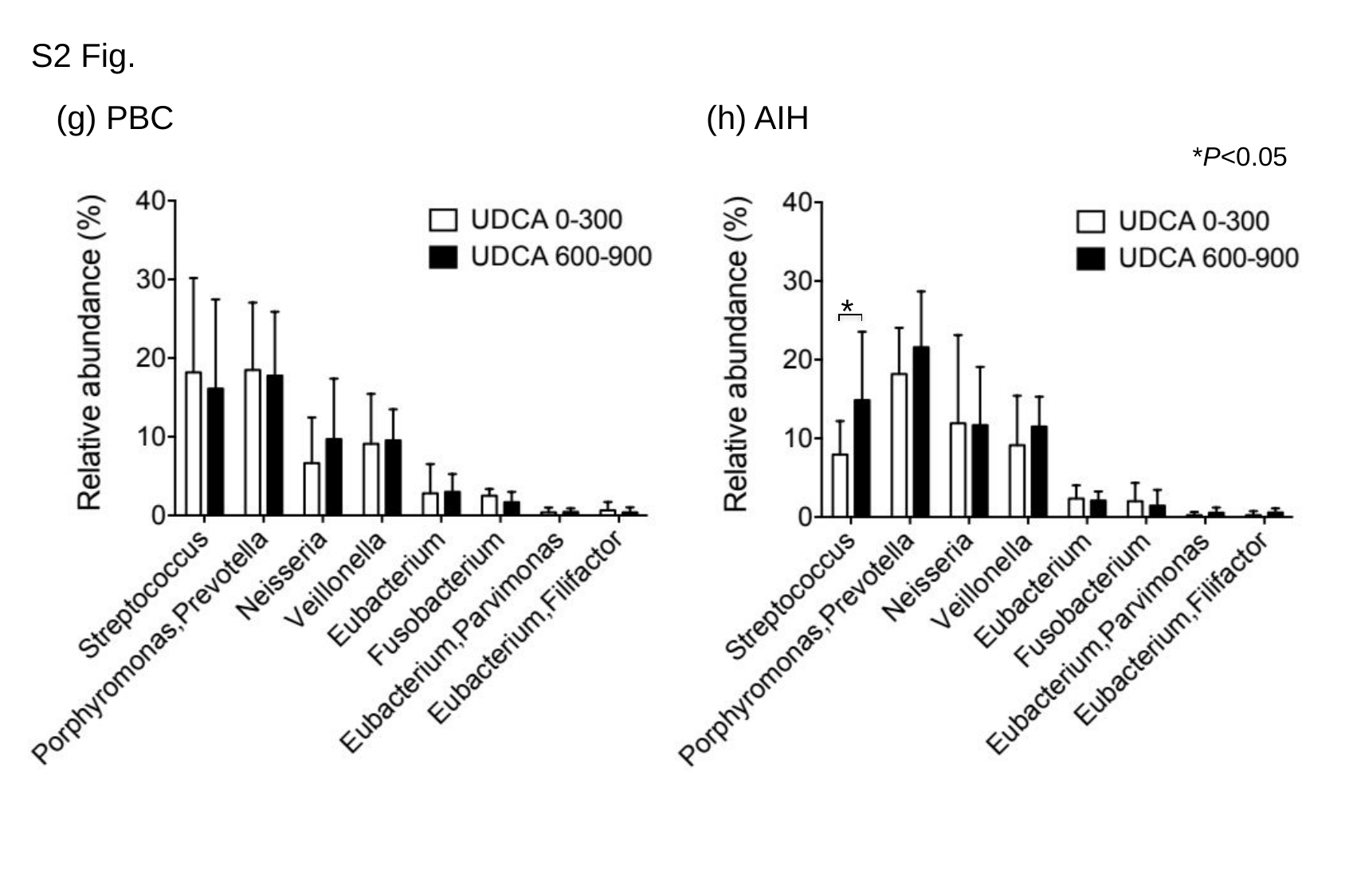

S2 Fig.
(g) PBC
(h) AIH
 *P<0.05
*

## Slide 5
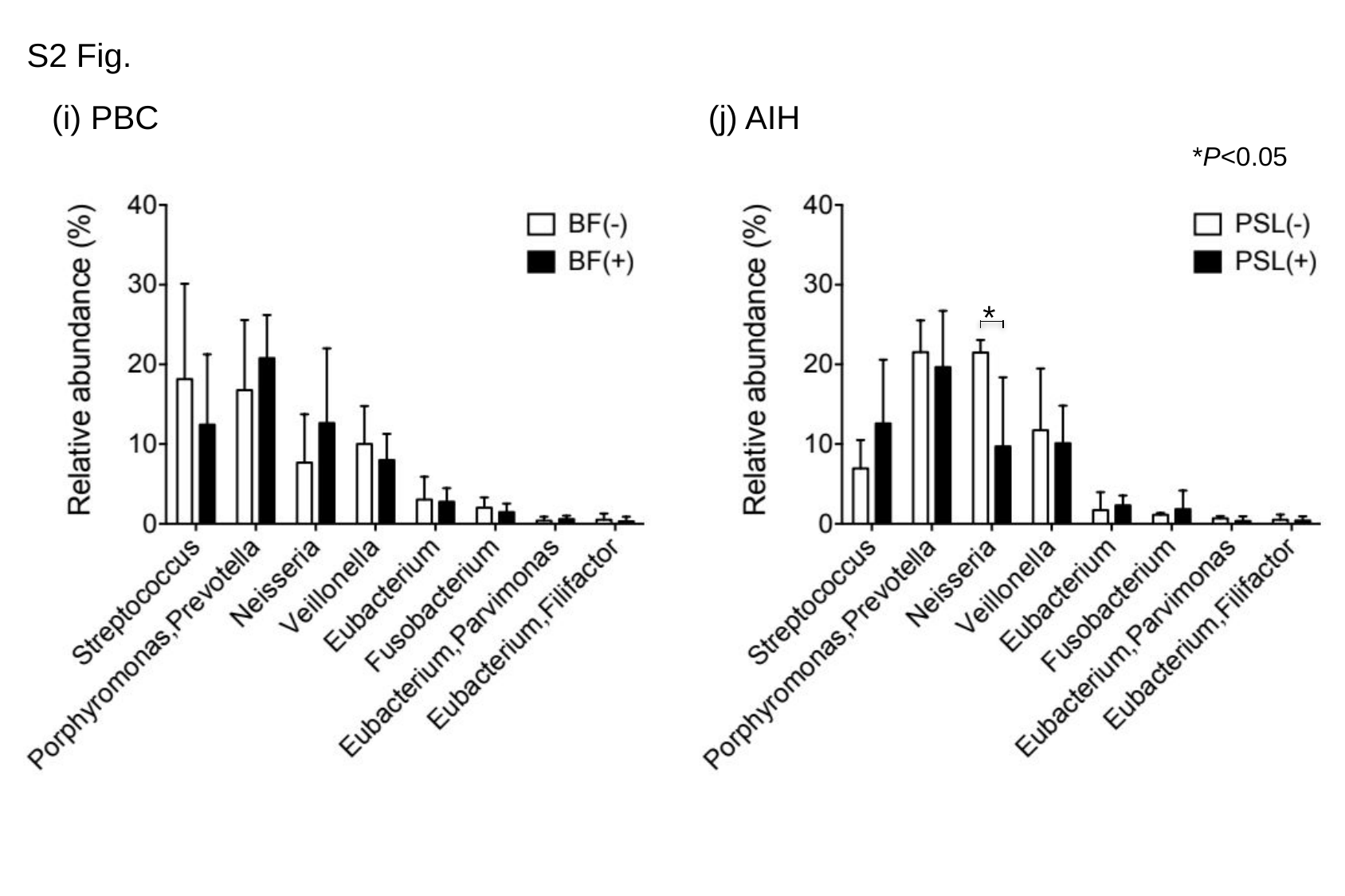

S2 Fig.
(i) PBC
(j) AIH
 *P<0.05
*
